# Hematological Correlations as Predictors of Disease Manifestations in Psychiatric Inpatients

**DOI:** 10.3390/nu17060959

**Published:** 2025-03-10

**Authors:** Maciej Domański, Anna Domańska, Sabina Lachowicz-Wiśniewska, Wioletta Żukiewicz-Sobczak

**Affiliations:** 1 Department of Nutrition and Food, Faculty of Medicine and Health Sciences, University of Kalisz (Calisia University), Nowy Świat 4, 62-800 Kalisz, Poland; maciej.domanski@interia.pl (M.D.); anna_domanska@onet.pl (A.D.); 2 Department of Biological Bases of Food and Feed Technologies, Faculty of Production Engineering, University of Life Science in Lublin, 13 Akademicka Street, 20-950 Lublin, Poland

**Keywords:** psychiatric patients, predictors of disease emission and remission, hematology

## Abstract

**Background/Objective.** Psychiatric disorders exhibit significant symptomatic and etiopathological heterogeneity, complicating diagnosis and treatment. Hematological parameters may serve as indicators of overall health and predictors of psychiatric symptom manifestation and remission, particularly in long-term hospitalized patients. This study evaluated hematological and biochemical markers, including vitamin B12, vitamin D, and glucose levels, to explore their potential role in psychiatric disorders and disease progression. **Methods.** This prospective observational study was conducted from 1 January to 31 December 2022, at the M. Kaczyński Neuropsychiatric Hospital in Lublin, following ethical guidelines. The study included 28 psychiatric inpatients (18 women, 10 men) diagnosed with mental and behavioral disorders (ICD-10: F03, unspecified dementia, and F06.2, organic delusional disorder) and 10 controls without psychiatric diagnoses. Blood samples from both groups underwent hematological and biochemical analyses. Statistical tests included the Shapiro–Wilk test, Kruskal–Wallis test, and Tukey’s multiple range test. **Results.** Psychiatric patients had significantly lower vitamin B12 (278.00 pg/mL vs. 418.50 pg/mL, *p* = 0.026) and severe vitamin D deficiency (3.00 ng/mL vs. 26.00 ng/mL, *p* < 0.001). Hematocrit levels were also lower (38.00% vs. 41.30%, *p* = 0.033), suggesting anemia risk. No significant differences in glucose levels were found. Reduced mean platelet volume and altered leukocyte subtypes suggested immune dysregulation. **Conclusions.** Nutritional deficiencies, particularly in vitamin B12 and D, play a critical role in psychiatric disorders. Routine screening and targeted supplementation should be integral to psychiatric care. Addressing these deficiencies may improve treatment outcomes, reduce symptom severity, and enhance patient well-being. Integrating metabolic and nutritional assessments into psychiatric practice is essential for advancing research and clinical management.

## 1. Introduction

Psychiatric and neurological diseases pose a significant health challenge, with an increasing number of individuals affected across various age groups. These conditions, characterized by complex etiologies, disrupt societal functioning and significantly diminish patients’ quality of life. Consequently, prevention has become a priority in addressing these disorders. The international literature increasingly focuses on analyzing etiological factors contributing to mental illnesses, highlighting prevention strategies such as maintaining a healthy lifestyle, a balanced diet, and regular physical activity, along with public education about these diseases [1].

A crucial aspect of current research is identifying early symptoms and predisposing factors for psychiatric and neurological disorders. Standard clinical evaluations of patients with these conditions often include hematological and biochemical blood tests. For instance, studies on schizophrenia and bipolar disorder patients have analyzed basic hematological parameters [1]. These tests provide insights into oxygen transport, tissue oxygenation, and iron levels, with hemoglobin (Hgb) and erythrocyte (RBC) counts playing a vital role. Epidemiological data underscore the issue of elevated blood glucose levels, particularly glycosylated hemoglobin (HbA1c), which correlates with reduced gray matter volume and depression [2,3]. Moreover, diet westernization—characterized by excessive consumption of processed foods—leads to nutrient deficiencies and, when combined with stimulants and physical inactivity, can result in severe health issues, including carcinogenesis [3,4]. Additionally, growing evidence links hyperhomocysteinemia to depressive disorders, dementia, Parkinson’s disease, cardiovascular conditions, and cerebrovascular diseases [5,6,7,8,9,10,11]. These findings emphasize the importance of integrating dietary and lifestyle interventions with medical approaches to mitigate the impacts of psychiatric and neurological diseases.

Therefore, the study aimed to evaluate hematological parameters used to assess the health and nutritional status of the body. Based on the determinations, the influence of the deficiency of examined hematological parameters (morphology with blood smear) and biochemical parameters, e.g., vitamin B12, vitamin D3, and glucose/glycemia levels, was identified on the occurrence and severity of psychotic symptoms. Additionally, the possibility of using nutritional interventions and supplementation as additional methods of antipsychotic treatment was also studied. An attempt was made to identify hematological correlations forecasting the manifestation of the disease in the blood of patients in locked wards.

## 2. Materials and Methods

The principle of the method was a prospective observational assessment of a clinical study type in which various physiologies and pathological parameters were evaluated in patients with psychiatric illnesses. The implemented research and determination of clinical, test, and laboratory parameters were carried out together with all other routine procedures in the treatment and monitoring of patients during hospitalization at the Psychiatric Care and Treatment Facility (Polish ZLOP) of the M. Kaczyński Neuropsychiatric Hospital in Lublin, following the ethical principles of working with patients.

The research was carried out as part of a project entitled: “Assessment of pro- and anti-health behaviors of a selected group of patients. Evaluation of peripheral blood morphology parameters and specific vitamins in patients with selected disorders of the nervous and circulatory systems” (Resolution No. 5/2021). It was performed in the period 1 January 2022–31 December 2022 at the Neuropsychiatric Hospital in Lublin in the locked ward.

### 2.1. Research Group

The inclusion criteria for the study were the diagnoses of mental and behavioral disorders according to the ICD [12], namely F03 (unspecified dementia) and F06.2 (organic delusional disorder) [Table 1]. Twenty-eight patients from the ward at the Psychiatric Care and Treatment Facility (ZLOP) of the M. Kaczyński Neuropsychiatric Hospital in Lublin, including 18 women and 10 men, were qualified for the study. This number included 16 patients with F03, among which there were 12 women and 4 men, and 12 with patients F06.2, including 6 women and 6 men. The study included patients staying in a psychiatric ward with a long-term diagnosis of the disease (F03, F06.2) and staying in the ward for many years, on average 3–6 years.

F03 Unspecified dementia. This category should be used when the general criteria for the diagnosis of dementia are met, but the specific type of dementia (F00.0-F02.9) cannot be determined. This code includes presenile and senile dementia NOS, presenile and senile psychosis NOS, and primary degenerative dementia NOS [12].

F06 other mental disorders due to brain damage or dysfunction and to physical disease [12].

F06.2 Disorders in which persistent or recurrent delusions dominate the clinical picture. The delusions may be accompanied by hallucinations, though with unrelated content. Some features suggestive of schizophrenia, such as bizarre delusions, hallucinations, or thought disorder, may be present. Diagnostic clues: in addition to the general criteria given in the introduction to F06, delusions (persecutory, of body change, jealousy, illness, death of the patient, or another person) should be diagnosed. Hallucinations, thought disorders, or isolated catatonic symptoms may be present. Consciousness and memory are not altered. The code includes delusional and delusional-hallucinatory organic conditions and schizophrenia-like psychosis of epilepsy [12].

### 2.2. Control Group

The control group consisted of patients from the outpatient clinic of the Primed Medical Centre in Lublin who did not have a diagnosed mental illness. The patients agreed to participate in the study, according to the project procedure, and were thus qualified to participate. The control group comprised 10 subjects (8 women and 2 men).

### 2.3. Research Methods

Blood samples were collected by qualified medical personnel during the patient’s stay in the ward of the M. Kaczynski Neuropsychiatric Hospital in Lublin. The blood samples from patients in the control group were taken during their visits to Primed Medical Centre after prior qualification by a primary care provider (PCP). The following blood tests were performed: (I) Hematology, including 5-DIFF morphology (RBC red blood cell count, HGB—hemoglobin concentration, MCV—mean corpuscular volume, MCH—mean corpuscular hemoglobin, MCHC—mean corpuscular hemoglobin concentration, RDW—red blood cell distribution width, PLT—platelet count, MPV—mean platelet volume, WBC—white blood cell/leukocyte count, NEU—neutrophil count, LYM—lymphocyte count, MON—monocyte count, EOS—eosinophil count, BAS—basophil count, NEU#—neutrophil percentage, LYM#—lymphocyte percentage, MON#—monocyte percentage, EOS#—eosinophil percentage, BAS#—basophil percentage); all the analyses were performed on a Pentra XL 80 instrument. (II) Immunology (Vitamin B12, Vitamin D); all the analyses were performed on a Cobas e411 instrument. (III) Glycaemia, on an instrument (patients of the M. Kaczyński Neuropsychiatric Hospital in Lublin) D+Blood Glucose Meter VGM 47 2AAA LR035, nr ser. SN 365A0000038/blood glucose level (patients of the Primed Medical Centre). Additionally, the following measurements were taken: body mass [kg], height [cm], and BMI.

Analyses were performed and authorized by laboratory diagnosticians. Medical staff (nurses on duty) took measurements of the body weight and height measurements. Interpretation of the analyses was performed by a medical doctor with a specialization in psychiatry, looking after the patients in the ward of the M. Kaczynski Neuropsychiatric Hospital in Lublin.

### 2.4. Statistics

The Shapiro–Wilk test was used to assess the normality of the distribution. The results were compared using the Chi-square and Kruskal–Wallis test with rank-biserial correlation (due to non-parametric distribution) and Tukey’s multiple range test. For all analyses, *p* < 0.05 was assumed. The STATISTICA 13.3 PL (StataCorp LP., Colparametramilege Station, TX, USA) program was used for the statistical analysis.

## 3. Results

The studies carried out were related to standard hematological diagnostics, the main object of which is blood, particularly its morphotic elements and the tissues in which hemopoiesis occurs. According to Tomaszewski [13], hematological laboratory tests enable the assessment of the volume of circulating blood and the three cellular systems: red blood cells, white blood cells, and blood platelets. Basic laboratory tests include quantitative evaluation of peripheral blood composition, morphological analysis of elements of blood cells, and morphological and biochemical differential tests [13]. The reference ranges of the hematological parameters assessed are shown in Table 2.

Hemoglobin concentration in the blood depends on the number of red blood cells and, to a lesser extent, on the average hemoglobin content of the erythrocyte. Increased hemoglobin concentrations are observed in polycythemia and water–electrolyte disorders of the dehydration type. In contrast, decreased concentrations are observed in anemias and states of overhydration (hyperhydration) [Table S1] [13]. The reference ranges of the hematological parameters assessed are shown in Table 2. A median of 12.70 was determined in the study group of patients (the test group) compared to 13.70 in the control group. The results were within the reference range. However, in the case of the patients in the study group, the lower limit of the reference range was determined, indicating the risk of anemia [Table 2].

Hematocrit defines the ratio of the volume of morphotic elements, mainly erythrocytes, to the volume of whole blood and is expressed as a relative index or as a percentage. The reference ranges of the hematological parameters assessed are shown in Table S1. An increase in hematocrit can be due to a direct increase in erythrocyte count, macrocytosis, or water–electrolyte disorders of the dehydration (hypohydration) type. In contrast, the decrease is associated with fewer red blood cells or water–electrolyte disorders of the overhydration (hyperhydration) type [Table 2] [13]. A median of 38.00 was determined in the study group of patients (the test group) compared to 41.30 in the control group. The results are within the reference range [Table 2].

Red blood cell count refers to 1 mm^3^ or 1 L. Reference values are shown in the table (Table S1). A physiological increase in red blood cell counts occurs in people staying in high altitude conditions and those performing strenuous physical work under hypoxic conditions. The number of erythrocytes increases in primary and secondary polycythemia and, relatively, in water-electrolyte disorders of the hypohydration type. A decrease, on the other hand, is observed in primary and secondary anemias. A relative reduction is present in overhydration (hyperhydration), while a physiological decrease occurs during pregnancy [13,14].

The median erythrocyte level in the study group (the test group) was 4.25 compared to 4.43 in the control group. The results are within the reference range. However, in the case of the patient group, the erythrocyte level was closer to the lower limit of the reference range, which may indicate an initial disorder towards anemia (Table 3).

The indices characterizing RBCs—mean corpuscular volume (MCV), mean corpuscular hemoglobin (MCH), and mean corpuscular hemoglobin concentration (MCHC)—were analyzed. MCV levels were closer to the upper limit of the reference range in the study group, suggesting a potential shift toward macrocytosis. In the study, for a group of patients (the test group), a median MCV level of 92.00 was determined compared to 90.10 in the control group. The results were within the reference range. However, in the patient group, the MCV level was closer to the upper limit of the reference range, which may indicate an initial disturbance toward anemia. MCH expresses the amount of hemoglobin in an erythrocyte in pg and is calculated from the hemoglobin concentration and the number of erythrocytes. The standard hemoglobin content in a blood cell is between 27 and 32 pg (91.7–2.0 fmol). A decrease is seen in iron-deficiency anemias and hypotonic overhydration states. An increase in MCH is observed in macrocytic anemias, especially in megaloblastic anemias [13,14].

In the study, for the group of patients (the test group), a median of 31.00 was determined for the MCH level compared to 30.40 in the control group. The results are within the reference range. However, in the patient group, the MCH level was closer to the upper limit of the reference range, which may indicate initial disturbances toward anemia [Table 2 and Table S1].

MCHC measures the saturation of red cells with hemoglobin and is calculated from hemoglobin concentration and hematocrit. The standard hemoglobin concentration in erythrocytes is approx. two times higher than in blood and is 32–38 g/dL for the blood cell mass (20–24 mmol/L). An increase in MCHC is observed in hereditary spherocytosis and states of hypertonic dehydration. A decrease is characteristic of iron-deficiency anemia and also occurs in water-electrolyte disorders of the hypotonic hyperhydration type [13,14].

In the study, for the group of patients (the test group), a median of 34.00 was determined for the MCHC level compared to 33.10 in the control group. The results are within the reference range. However, in the patient group, the MCHC level is closer to the upper limit of the reference range, which may indicate an incipient disorder associated with a hypertonic dehydration state [Table 2 and Table S1].

On the other hand, a median of 8.55 was determined for the MPV level in the study group (the test group) compared to 10.45 in the control group. The results were within the reference range. However, in the patient group, the MPV level was closer to the lower limit of the reference range, which may indicate an initial disturbance in managing vitamin B and folic acid, which are essential for platelet production [Table 2 and Table S1].

WBCs include granulocytes, lymphocytes, and monocytes. Physiological leukocytosis (an increase in WCC) is observed after high physical exertion, under stress, in pregnant women, after childbirth, and in newborns. An increase in white blood cell count is a non-specific symptom of many diseases. Leukocytosis occurs in localized and generalized inflammation, in some infectious diseases, in poisoning, after trauma and surgeries, in some leukemias, in post-hemorrhagic conditions, during treatment with corticosteroids, adrenaline, lithium compounds, and in many other diseases. Leukocytosis is also observed in compulsive smokers. A decrease in the number of white blood cells in the blood (leukopenia) occurs in certain infectious diseases, especially in viral diseases (hepatitis and influenza), over the course of treatment with cytostatic and antimetabolic drugs, after irradiation with ionizing radiation, and in poisoning with certain chemical compounds. In general, the causes of leukopenia include impaired granulopoiesis or lymphopoiesis resulting from primary or secondary changes in the bone marrow or lymph nodes, inhibited maturation of leukocytes and their release into the blood from tissue reservoirs, increased peripheral consumption of white blood cells, especially in autoimmune diseases. Considering the various forms of white blood cells, it is diagnostically essential to diagnose leukocytosis or leukopenia and determine which leukocyte population is affected [Table S1] [13,14]. The study group’s median WBC count was 5.94 × 10^3^/µL compared to 6.60 × 10^3^/µL in the control group, closer to the lower limit of the reference range, potentially indicating early leukopenia [Table 2].

Lymphocyte and monocyte counts in the study group were within normal limits, while eosinophil levels were elevated, indicating possible allergic or parasitic conditions. Basophil levels were at the upper limit, suggesting potential endocrine or allergic disturbances [Table 2]. In peripheral blood smears of healthy people, only mature forms of leukocytes are observed, with a small percentage of granulocytes with band nuclei. The changes may consist of an increase or decrease in the number of specific forms of leukocytes or the appearance in the peripheral blood of precursor cells of the granulopoietic or lymphopoietic series or other abnormal cells. An increase in the percentage of neutrophilic granulocytes (neutrophilia) is most often caused by acute infection, especially with pyogenic bacteria. The number of granulocytes also increases in people treated with corticosteroids and in adrenal hyperactivity. In addition, neutrophilia can be caused by hypersecretion of adrenaline, e.g., in stressful situations, trauma, burns, etc. The condition is also observed after loss of large amounts of blood, in malignant diseases, in proliferative diseases of the bone marrow, and some poisonings, e.g., with heavy metals or carbon monoxide. An increase in the percentage of granulocytes leads to leucocytosis. On the other hand, eosinophilia, i.e., an increase in the number of acidophilic granulocytes, is a symptom characteristic of parasitic and allergic diseases. It also appears in skin, pituitary insufficiency, and hematological diseases, e.g., Hodgkin’s lymphoma. Eosinophilia may accompany neoplastic diseases. The number of basophilic granulocytes increases non-specifically in polycythemia and chronic myeloid leukemia. Lymphocytosis (an increase in the percentage of lymphocytes) is a physiological sign in infants and children up to 3–4 years of age. An increase in the number of lymphocytes is also observed in certain infections (influenza, pertussis, salivary gland inflammation, and brucellosis), mononucleosis, and infectious lymphocytosis, as well as in proliferative diseases (lymphomas), macroglobulinemia, and chronic lymphocytic leukemia. An increase in the percentage of monocytes (monocytosis) occurs in infections with protozoa and certain types of Gram-negative bacteria (rickettsiae), in chronic infections (tuberculosis, chronic endocarditis, and brucellosis), and in infectious mononucleosis. Sometimes, it also occurs in liver disease. A decrease in the percentage of specific white cells, often associated with general leukopenia, may be due to the inhibition of granulopoiesis or lymphopoiesis (vitamin B12 or folic acid deficiency, and radiation damage to the bone marrow), impaired release of granulocytes or lymphocytes from the bone marrow or organs involved in hemopoiesis, or selective excessive destruction of white cells in the circulatory system by antibodies or in blood cells removal organs. A decrease in the percentage of lymphocytes and acidophilic or basophilic granulocytes may accompany neutrophilic diseases. Granulopenia is also observed in some acute myeloid leukemias, in some infectious, viral, bacterial diseases (typhoid fever, malaria), and after using cytostatic and antiemetic drugs [13,14].

Parameters within the reference range were recorded for lymphocytes, monocytes, and neutrophils. A median of 2.27 (36%) was determined for the lymphocyte level in the study group (the test group) compared to 1.83 (30%) in the control group. The median for the monocyte level was 0.45 (7.00%) compared to 0.46 (7.75%) in the control group. On the other hand, the median for the neutrophil level was determined at 3.18 (52.50%) compared to 3.45 (59.65%) in the control group. In the study group of patients (the test group), the median for eosinophil level was determined at 0.22 (4.00%) compared to 0.11 (2.25%) in the control group. The results are within the reference range. However, an increase toward the upper limit of the reference range was observed in the study group, which may indicate initial changes toward parasitic and allergic diseases. Such an increase also appears in skin diseases, pituitary insufficiency, and hematological diseases. A median of 0.03 (1.00%) was determined for the basophil levels in the patient group (the study group) compared to 0.04 (0.50%) in the control group. The results are within the reference range. However, the upper limit of the reference range was observed in the study group, which may indicate incipient changes toward endocrine diseases, allergic diseases, or diabetic conditions, among others.

A physiological increase in platelet count is observed in infants 3 to 12 months of age, in women after childbirth, and, together with the increase in erythrocytes, in people staying at high altitude conditions (hypoxia). Trobocythaemia, i.e., an increase in platelet count, is seen after surgery, after splenectomy, in polycythemia vera, chronic myeloid leukemia (especially in the early stages), and megakaryocytic leukemia. Decreased platelet count, i.e., thrombocytopenia, can be caused by impaired bone marrow thrombopoiesis, increased spleen platelet breakdown, and increased consumption or destruction in autoimmune diseases [13,14].

Platelet count, essential for coagulation, was slightly lower in the study group (median 223.5 × 10^3^/µL) compared to the control group (236.5 × 10^3^/µL), both within reference ranges [Table 2 and Table S1] [13,14]. Reduced mean platelet volume (MPV) in the study group suggests potential vitamin B12 or folic acid deficiencies, which are crucial for platelet production.

Vitamin B12 and folic acid are essential for forming red and white blood cells and all body cells. It synthesizes deoxyribonucleic acid (DNA), amino acids, and bone marrow proteins with folic acid. It plays an important role in the metabolism of carbohydrates and fats, transmethylation (transfer), and synthesis of methyl groups (-CH3). Vitamin B12 is essential for the normal nervous system and gastrointestinal tract function. It is used in higher doses in Addison’s and Biermer’s disease, in anemias, in polyneuritis, and in liver disorders. Vitamin B12 deficiency causes the inhibition of cell maturation and proliferation, especially of red blood cells, resulting in pernicious (megaloblastic) anemia, mucosal atrophy (achlorhydria), and degenerative changes in the spinal cord and peripheral nerves. Degeneration in the peripheral and central nervous system is caused by impaired synthesis of choline, which is part of the phospholipids that build the myelin sheaths of nerves. Vitamin B12 deficiency can occur due to malabsorption in the intestine. A glycoprotein, called Castle’s factor, secreted by the gastric mucosa, is required to absorb vitamin B12 properly. This glycoprotein binds the vitamin in the presence of calcium ions. The resulting complex is absorbed by the small intestine. The complex combines with serum protein in the blood and is distributed throughout the body in this form. Part of the vitamin accumulates in the liver (approx. 1 µg/1g tissue). Very frequently, deficiencies of this vitamin are observed in people following plant-based diets. The primary sources of the vitamins are the liver and kidneys, in smaller amounts, meat, milk and milk products, egg yolks, and, in marginal amounts, the bacterial flora in the digestive tract also produces it. In the study group of patients (the test group), the median of 278.00 was determined for the vitamin B12 levels in blood compared to 418.50 in the control group. The results are within the reference range. However, the levels of the vitamin measured in the study group were almost half that of the control group. Hence, the level of vitamin B12 obtained in the test group is closer to the lower limit of the reference range, which may indicate a risk of deficiencies of foodstuffs rich in this nutrient [Table 2 and Table S1] [15].

Three compounds belonging to the steroids, which exhibit anti-rocket properties, are referred to as Vitamin D. These compounds are cholecalciferol—vitamin D3, ergocalciferol—vitamin D2, and 25-hydroxycholecalciferol. The human body synthesizes cholecalciferol from provitamin (7-dehydrocholesterol present in the skin) under the influence of ultraviolet rays, while ergocalciferol is synthesized from ergosterol found in plant products (yeast, mushrooms). The active form of vitamin D is transferred to various organs (mainly to the liver), where it is deposited in sufficient quantities, even for several weeks. In addition, some vitamin D is deposited in adipose tissue. The functions of vitamin D in the body are related, among other things, to the functioning of the skeletal system, as it enhances the resorption of calcium and phosphate in the intestines. This vitamin is essential for calcium transport processes. It plays a vital role in regulating the body’s calcium-phosphate balance by influencing the development and mineralization of bone tissue. It influences the secretion of parathormone produced by the parathyroid glands. It is a hormone-like vitamin, as its action in many processes is similar to that of steroid hormones. Vitamin D is absorbed with food in the small intestine in the presence of bile acids, fatty acids, and triglycerides. It is then transported with chylomicrons through the lymphatic system into the bloodstream. The form of vitamin D formed in the skin, as well as the form absorbed from the gastrointestinal tract, enters the liver, where it is hydroxylated by the attachment of the OH group (at pos. 25), thus becoming the primary form of vitamin D—25 (OH)D. After combining with protein, this metabolite enters the kidneys. Here, it undergoes further hydroxylation (at position 1), transforming into the most hormonally active form of vitamin D. This form influences the absorption of calcium in the intestine, is involved in the mobilization of calcium from the bones, and increases calcium concentration in the blood. Vitamin D deficiency is very dangerous. In infants and young children, it causes rickets. In adults, especially in pregnant and lactating women, deficiency of this vitamin causes decalcification and softening of the bones (osteomalacia), while in the elderly—osteoporosis, i.e., demineralization, deformation of the bones, decreased bone density, porosity, and fragility of the skeletal system. When vitamin D levels are low, calcium is not fully absorbed from food. Low vitamin D levels may be caused by impaired absorption of fatty acids due to low bile acid levels. The body derives about 80% of vitamin D from skin synthesis, with the remaining amount from the diet. The primary sources of vitamin D3 in food are dairy products, such as eggs, butter, cheese, and milk. However, the most significant amounts of this vitamin are found in fish and marine mammal fats (fish and marine mammal oil). In addition, substantial amounts are found in liver, margarine, and modified infant milk enriched with this component. Yeast and fungi are sources of vitamin D2 [Table 2] [15,16,17]. In the study group of patients (the test group), the median of 3.00 was determined for the vitamin D levels in blood among the study groups compared to 26.00 in the control group. In the test group, the results are not within the reference range. Severe deficiencies of this vitamin were observed. However, the levels of the vitamins tested by the representatives of the control group were also not satisfactory as they were insufficient. Polish population is at risk of vitamin D deficiency in the autumn-winter and winter-spring months. Hence, a well-balanced diet, as well as supplementation, is recommended. However, vitamin D deficiencies in the study group are very advanced compared to the control group. Long-term vitamin D deficiency can lead to demineralization, skeletal deformation, decreased bone density, porosity, and fragility of the skeletal system. When vitamin D levels are low, calcium is not fully absorbed from food. This may be due to impaired absorption of fatty acids caused by low bile acid levels [14].

Blood glucose levels were assessed as an indicator of metabolic health. Their concentration is precisely regulated, mainly by hormones, and in the healthy state, it ranges from 3.90 to 9.90 mmol/dL (70–180 mg/dL). This range applies to values occurring on an empty stomach, after several hours of starvation, and after carbohydrate-rich meals. In whole blood, glucose concentration is approximately 10 percent lower. Glucose levels in fasting blood or serum above 6.6 mmol/L (120 mg/dL) are treated as hyperglycemia. High concentrations (500 mg/dL and more) are mainly observed in uncompensated diabetes, depending on the severity of the condition. Hyperglycaemia resulting from other causes is generally moderate and may be triggered by hyperactivity of the pituitary, thyroid gland, and adrenal glands or by stimulating the sympathetic-adrenal system (stress). It sometimes occurs in infectious diseases, neurological diseases, pancreatitis, and pancreatic cancer. Glucose concentrations increase in patients undergoing general anesthesia. Even overt diabetes with a mild course does not necessarily cause hyperglycemia. Hence, an average glucose level in fasting blood does not rule out a disorder of carbohydrate metabolism. In contrast, a drop in blood glucose below 2.2 mmol/L (40 mg/dL) or serum glucose below 2.5 mmol/L (45 mg/dL) manifests hypoglycemia. A drop in the glucose concentration below 40–30 mg/dL, especially occurring rapidly, leads to clinical symptoms, cerebral hypoxemia, and hypoglycaemic coma. There are many possible causes of hypoglycemia; among them are the following: prolonged starvation, especially combined with physical exertion; insulin hypersecretion, e.g., in pancreatic insulinoma or pancreatic islet hyperplasia; deficiencies of hormones with effects antagonistic to insulin, e.g., glucocorticosteroids in adrenal insufficiency; a more severe form of hypothyreosis; and some liver diseases, especially glycogen storage diseases. In practice, the use (overdose) of glucose-lowering drugs such as insulin or sulfonylurea derivatives is often the cause of this condition. A decrease in glucose concentration occurs 2-8 h after alcohol consumption. In some individuals, hypoglycemia induced by ingestion of monosaccharides (such as glucose, galactose, and fructose) or leucine is observed. It occurs 5–6 h after consumption [13,14]. The study group median was 92.5 mg/dL, within the reference range but slightly higher than the control group (89.5 mg/dL). Elevated glucose and HbA1c levels are associated with reduced gray matter volume and depression [3]. Long-term hyperglycemia can result from stress, endocrine disorders, or diabetes [Table 2].

Peripheral blood smears revealed erythrocyte size, shape, and hemoglobin content abnormalities. Microcytosis (small RBCs) is characteristic of iron-deficiency anemia, while macrocytosis and megalocytes indicate vitamin B12 or folic acid deficiencies. Hypochromic cells (reduced hemoglobin) were observed in iron deficiency anemia [Table 2 and Table S1] [13,14].

In WBC morphology, granulocyte and lymphocyte abnormalities were noted. Neutrophilia (increased neutrophils) suggests acute infection or stress, while eosinophilia points to allergic or parasitic conditions [Table S1] [13]. Monocytosis in some patients may indicate chronic infections or liver disorders [Table 2].

The findings underscore the importance of regular hematological assessments in identifying early indicators of anemia, infections, or nutrient deficiencies. The significant deficiencies in vitamin D and vitamin B12 among study participants highlight the need for targeted interventions, including dietary education and supplementation. Maintaining adequate glucose levels through lifestyle modifications is critical to preventing metabolic and neurological disorders.

This study reinforces the interconnected nature of hematological parameters, nutrient status, and overall health. Early detection and intervention can mitigate the risks of chronic diseases, improving patient outcomes.

Furthermore, there were significant (*p* > 0.05) differences between the test and control groups regarding anthropometric data, i.e., age, weight, and height. BMI did not indicate significant differences between the two study groups of patients [Table 3].

Significant (*p* > 0.05) differences were found between the test and control groups regarding biochemical results, i.e., vitamin B12, vitamin D3, hematocrit, RDW-SD, MPV, and eosinophils (%). However, the other parameters studied showed no significant differences between the two researched groups [Table 4].

No mutual effect was shown for the parameters such as age and gender among the study groups. Thus, no significant differences were found concerning vitamin B12 and D content, BMI parameters, body weight, waist circumference, and glycemia, which indicates no reciprocal effect.

**Figure 1:**
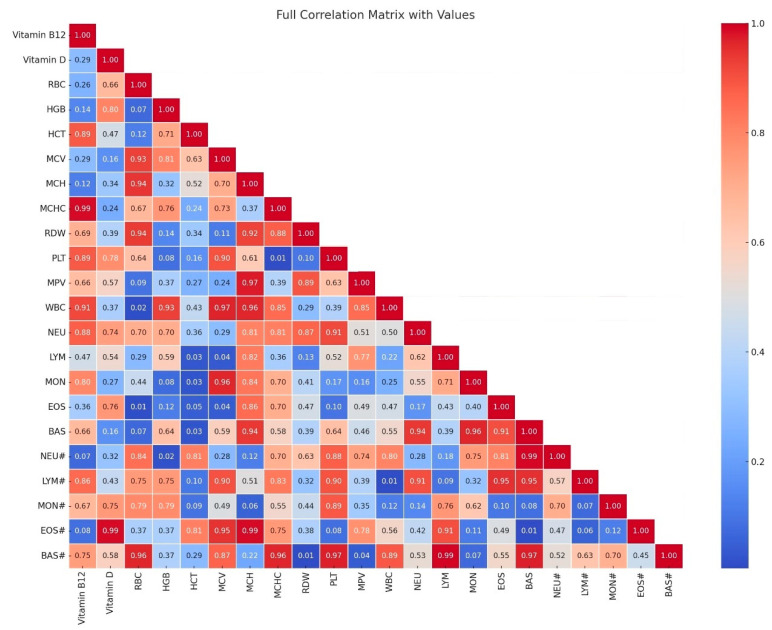
A full correlation matrix with values. Legend: Red Blood Cell (RBC), Hemoglobin (HGB), Hematocrit (HCT), Mean Corpuscular Volume (MCV), Mean Corpuscular Hemoglobin (MCH), Mean Corpuscular Hemoglobin Concentration (MCHC), Blood Cell Volume (RDW), Platelets (PLT), Mean Platelet Volume (MPY), white Blood Count (WBC), Neutrophils (NEU), Lymphocytes (LYM), Monocytes (MON), Eosinophils (EOS), Basophils (BAS), Neutrophils % (NEU#), Lymphocytes % (LYM#), Monocytes % (MON#), Eosinophils % (EOS#), Basophils % (BAS#).

In our studies, we noted a strong correlation (*p* > 0.05; high correlation coefficient) between the parameters erythrocytes (RBC) and hemoglobin (HGB), as well as hematocrit (HCT) (r = 0.88 and 0.89), between hemoglobin (HGB) and hematocrit (HCT) (r = 0.98). In turn, between the mean corpuscular volume (MCV) and the mean corpuscular size (MCH), the correlations were noted at the level of (r = 0.94). The correlation between neutrophils in percentage (NEU) and lymphocytes in percentage (LYM) was the level of (r = 0.92). Correlations between leukocytes (WBC) and neutrophils (NEU#) were at the level of (r = 0.88), and in turn, the correlation between leukocytes (WBC) and basophils (BAS#) was at the level of (r = 0.83). On the other hand, the parameters with moderate correlation were noted between lymphocytes (LYM#) and monocytes (MON#), and monocytes in percentage (MON), and these were levels (r = 0.72 and 0.76). Between leukocytes (WBC), lymphocytes (LYM#), and monocytes (MON#), correlations were calculated at the level (r = 0.71 and 0.61). The relationships considered next had decreasing trends: between vitamin D and platelets (PLT) and noted level (r = 0.61), leukocytes (WBC) -level (r = 0.72), neutrophils (NEU#) level—(r = 0.58), lymphocytes (LYM#) level (r = 0.52), monocytes (MON#) level (r = 0.63), and basophils (BAS#) level (r = 0.73). The lowest correlations were noted between vitamin B12 and vitamin D (r = 0.48), and between monocytes (MON#) level (r = 0.50) and basophils [Figure 1].

### Summary of Key Laboratory Findings in Psychiatric vs. Control Groups

The laboratory analysis revealed significant differences in key nutritional markers between psychiatric patients and the control group. The most prominent findings include significantly lower vitamin B12 levels (278.00 pg/mL in psychiatric patients vs. 418.50 pg/mL in controls, *p* = 0.026) and severe vitamin D deficiency (3.00 ng/mL in psychiatric patients vs. 26.00 ng/mL in controls, *p* < 0.001) in the psychiatric group. These deficiencies highlight a potential role of vitamin insufficiencies in the etiology and progression of psychiatric disorders, particularly in long-term hospitalized patients.

Additionally, hemoglobin (HGB) and hematocrit (HCT) levels were lower in psychiatric patients (12.70 g/dL vs. 13.70 g/dL and 38.00% vs. 41.30%, respectively), suggesting an increased risk of anemia. While glucose levels were comparable between the groups (92.50 mg/dL vs. 89.50 mg/dL, *p* = 0.145), minor alterations in hematological parameters, including a decreased platelet count (PLT) and variations in leukocyte subtypes, were observed, indicating potential links between immune dysregulation and psychiatric disorders.

Taken together, these findings emphasize the need for routine nutritional assessment and targeted supplementation in psychiatric care, particularly for long-term inpatients, where metabolic and nutritional imbalances may exacerbate symptoms and contribute to disease progression. Addressing these deficiencies through dietary adjustments and supplementation may play a crucial role in improving overall health outcomes in psychiatric populations.

## 4. Discussion

The study examined hematological and biochemical parameters in patients from a psychiatric ward, comparing a test group with a control group to explore potential links between hematological changes and psychiatric disorders. The test group had a median hemoglobin level of 12.70 g/dL compared to 13.70 g/dL in the control group (*p* = 0.239). While within the normal range, the test group values approached the lower limit, indicating a potential risk of anemia. These findings align with Memić-Serdarević et al. [1], who observed similar trends in patients with bipolar disorder (BD) and schizophrenia (SCH). At the same time, Okruszek et al. [18] found that patients with SCH had higher mean HGB and erythrocyte counts. Hematocrit levels in the test group were 38.00%, compared to 41.30% in the control group (*p* = 0.033), and erythrocyte counts were 4.25 × 10^6^/µL versus 4.43 × 10^6^/µL (*p* = 0.082). While both parameters were within reference ranges, the test group results were closer to the lower limits, suggesting early disturbances toward anemia. Memić-Serdarević et al. [1] similarly reported reduced erythrocyte levels in psychiatric patients.

Mean corpuscular volume (MCV), mean corpuscular hemoglobin (MCH), and mean corpuscular hemoglobin concentration (MCHC) were higher in the test group than in the control group, with medians of 92.00 fl, 31.00 pg, and 34.00 g/dL, respectively. These values, while normal, approached upper reference limits, potentially indicating early macrocytic changes. Platelet counts were slightly lower in the test group (223.50 × 10^3^/µL) than in the control group (236.50 × 10^3^/µL), which is consistent with potential vitamin B12 or folic acid deficiencies [13]. According to Memić-Serdarević [1], patients with BD had higher MCV values but, at the same time, lower mean RBC values. The MCH results of the patients in our experiment were similar to those in the Memić-Serdarević et al. [1] study. Mean platelet volume (MPV) was significantly reduced in the test group (8.55 fl) compared to the control group (10.45 fl). MPV reductions suggest disturbances in platelet metabolism, possibly related to serotonin transporter dysfunction or inflammatory processes [19,20,21,22,23]. Some studies have shown an association between platelet counts and serotonin transporters, which are, in turn, associated with an increased risk of cardiovascular disease in people with depression and anxiety [19]. These findings align with studies linking MPV changes to anxiety and depression [24].

Parameters for the morphotic assessment of red blood cells were also determined. In the study group of patients (the test group), the median of 13.00 was calculated for the RDW-SD level compared to 14.80 in the control group. The results are within the reference range. This indicator provides information on the difference between the volumes of individual red blood cells. Together with other morphological indices, it is a valuable parameter in finding causes of anemia. In our study, we observed reduced red blood cell parameters in the patients from the test group, which is consistent with many experiments described in the literature that support the hypothesis that low red blood cell parameters may contribute to the risk of developing some psychiatric disorders, such as depression [18].

Such correlations are coherent with previous studies showing the same results with dehydration occurring due to acute psychosis [20]. Inflammatory processes are thought to play a significant role in the aetiopathogenesis of BD, and there is some suggestion that inflammatory cytokines in the systemic circulation inhibit erythropoiesis, which leads to anemia of inflammation, known as anemia of chronic disease [21]. Hochman et al. [22] found that patients during a depressive episode had higher levels of HGB and HCT and lower levels of HGB and HCT during manic episodes [22]. By comparing the haemogram values of patients in the test group and the control group, it was demonstrated that the proportion of inflammatory cells changed during manic episodes, which indeed supports the hypothesis of activation of inflammatory processes during such episodes.

From the literature, it is known that platelet volume depends on increased platelet activation or production and the presence of immature forms in the peripheral blood. In turn, platelet activation and release from granules leads to a decrease in platelet volume, which is associated with a reduction in peripheral platelet count, and therefore, bone marrow activity and the presence of immature forms in the blood are inevitable. This increases PDW [10,23]. The studies on platelet disorders in people with anxiety indicate damage to the serotonin transporter. In our study, we observed a significant reduction in MPV, suggesting a possible disturbance in the metabolism of serotonin and its transporters. This fact may be a potential indicator for therapy in these patients. Similar results were observed in studies by other authors [1]. According to Atagun et al. [24], improvements in this parameter occur after lithium therapy [19,24].

Leukocyte levels were within the reference ranges but lower in the test group (5.94 × 10^3^/µL) compared to the control group (6.60 × 10^3^/µL), indicating early leukopenia. However, in the patient group, leucocyte levels were closer to the lower limit of the reference range, which may indicate incipient disorders toward leukopenia. The lymphocyte levels were within normal limits, ranging from 2.27 × 10^3^/µL (36%) in the sick patient group to 1.83 × 10^3^/µL (30%) in the control group. Similarly, monocyte levels were within the reference range, ranging from 0.45 × 10^3^/µL (7.00%) in the sick patient group (the test group) to 0.46 × 10^3^/µL (7.75%) in the control group, just like the neutrophil levels, which ranged from 3.18 × 10^3^/µL (52.50%) in the patient group (the test group) to 3.45 × 10^3^/µL (59.65%) in the control group. The median of the eosinophil level was determined to be 0.22 × 10^3^/µL (4.00%) in the patient group (the test group) compared to 0.11 × 10^3^/µL (2.25%) in the control group. The results are within the normal range. However, an increase toward the upper limit of the reference range was observed in the test group, which may indicate initial changes toward parasitic and allergic diseases. This relationship also appears in skin diseases, hypofunction of the pituitary gland, and hematological diseases. Another interesting fact was the relationship observed in the case of basophil levels. In the study group of sick patients (the test group), the median was 0.03 × 10^3^/µL (1.00%) compared to 0.04 × 10^3^/µL (0.50%) in the control group. The results are within the reference range. However, the upper limit of the reference range was observed in the study group, which may indicate incipient changes toward endocrine diseases, allergic diseases, or diabetic conditions [1,25]. Eosinophil levels were elevated in the test group (0.22 × 10^3^/µL; 4%) compared to the control group (0.11 × 10^3^/µL; 2.25%), indicating potential allergic or parasitic conditions. Lymphocytes, monocytes, and neutrophils were similarly within normal limits but showed slight differences between groups, supporting inflammatory hypotheses in psychiatric disorders. Basophil levels also approached the upper limit of normal in the test group, suggesting endocrine or allergic influences [26].

Research links inflammatory processes with BD and SCH, suggesting cytokines may inhibit erythropoiesis, leading to chronic disease anemia [21]. Studies also report that elevated neutrophil counts correlate with brain matter loss in psychotic disorders, potentially due to immune dysregulation [27]. Our findings of altered white cell distributions support these observations. At this point, it is worth noting the study by Núñez et al. [27], conducted in 2019, concluded that white blood cell values are related to brain volume and/or clinical picture. We know from the literature that neuroradiological studies have confirmed changes in brain matter in patients with psychotic disorders, confirming the above. From the literature, we know that in the group of patients with a first psychotic episode, neutrophil values were associated with reduced gray matter volume and increased cerebrospinal fluid volume (CT scan analysis). Studies by Núñez et al. [27] indicate that brain tissue loss is associated with neutrophil concentrations in psychotic disorder, which supports the hypothesis of immune system deregulation. The cited researchers concluded that elevated neutrophil count is associated with a more severe clinical picture, which is an apt indicator in evaluating the clinical picture of SCH and suggesting new therapeutic approaches. Therefore, further research is needed to confirm these relationships related to inflammatory factors. We also agree with the assertion of other researchers that it would be helpful to study the comparison between the acute phase and the remission phase to understand whether inflammatory changes can be considered a symptom or, more specifically, a feature of a specific psychiatric disorder. Furthermore, it is necessary to investigate the effect of psychopharmacological treatment on inflammatory features.

Glucose levels in the test group (median 92.50 mg/dL) were within reference ranges but slightly higher than in the control group (89.50 mg/dL). McAvoy et al. [28] reported higher scores in psychiatric patients. Based on the findings from the literature, the relationship between glucose tolerance and psychiatric disorders is quite controversial. Most articles, originating mainly from the US, display an increased prevalence of diabetes among psychiatric patients or no association between psychiatric disorders and diabetes. Our results and the data provided by McAvoy et al. [28] and Ohwovoriole et al. [29] do not support the suggestion that there is an increased impairment of glucose tolerance in people with psychiatric disorders. Metabolism of ingested glucose in psychiatric patients is probably related to some antipsychotic drugs, in particular chlorpromazine. Hence, there is a need to study a more significant set of people, especially those who have yet to begin the treatment with antipsychotic medications. More research in this area will help to define further the relationship between the prevalence of glucose intolerance and psychiatric disorders.

Vitamin B12 levels in the test group (278.00 pg/mL) were significantly lower than in the control group (418.50 pg/mL) but within normal ranges. Deficiencies in vitamin B12 can impair erythropoiesis, leading to anemia and neurological symptoms. Educational initiatives and guidelines for early detection and supplementation of vitamin B12 in high-risk populations are crucial [30].

Severe vitamin D deficiency was noted in the test group (median 3.00 ng/mL) compared to insufficient levels in the control group (26.00 ng/mL). Vitamin D is key in bone health, immune function, and brain activity [31]. Deficiencies in psychiatric patients may contribute to disease progression, highlighting the need for dietary supplementation, particularly in regions with limited sunlight.

Hematological and biochemical changes observed in the test group support the role of inflammation, nutrient deficiencies, and metabolic disruptions in psychiatric disorders. Reduced red blood cell parameters and altered white cell distributions reflect underlying inflammatory and immune processes. Monitoring hematological profiles could guide early interventions, such as anti-inflammatory therapies and micronutrient supplementation, to improve outcomes in psychiatric patients [32].

Further research should explore longitudinal changes across disease phases and treatment responses to establish causative links and refine therapeutic approaches.

Furthermore, based on our study, we agree with the existing consensus that clinical symptoms need more attention when diagnosing vitamin B12 deficiency. Laboratory markers of vitamin B12 can support this diagnosis. The severity of clinical symptoms, the causes of vitamin B12 deficiency, and the treatment goals determine decisions regarding the intake route and dosage of vitamin B12 therapy [30].

Furthermore, as mentioned above, we agree with the need for research on the correlation between low 25(OH)D levels in serum and the prevalence of the diseases mentioned. It is essential to consider the clarity of the consensus on whether 25(OH)D measurement in serum can be clinically useful as a biomarker for diagnosing, prognosis, and predicting treatment response in neurodegeneration, psychiatric diseases, and immunological disorders [31,32].

The observed hematological correlations in this study provide valuable insight into the biological underpinnings of psychiatric disorders. Given the increasing evidence linking systemic inflammation, oxidative stress, and immune dysregulation to psychiatric conditions such as schizophrenia and mood disorders, the role of hematological markers in predicting disease manifestation and severity warrants further investigation. Previous studies have indicated that alterations in red and white blood cell indices may reflect inflammatory processes, nutritional deficiencies, and metabolic imbalances frequently observed in psychiatric patients [1,27]. In our study, strong correlations between hemoglobin, hematocrit, and red blood cell count (r = 0.88–0.98) suggest a potential association with anemia. This condition has been previously linked to cognitive impairment and psychiatric symptoms, particularly in elderly and chronically hospitalized patients [28]. Additionally, moderate correlations observed between vitamin D and immune parameters such as WBC, NEU, and MON (r = 0.52–0.73) align with findings that indicate the immunomodulatory role of vitamin D in neuropsychiatric disorders, including schizophrenia and major depressive disorder [31]. Moreover, the negative correlation between vitamin B12 and vitamin D (r = 0.48) supports previous evidence that vitamin deficiencies may contribute to neurodegenerative processes, cognitive dysfunction, and depressive symptomatology [30]. These findings underscore the necessity of routine hematological assessments in psychiatric patients as a means of identifying early physiological disturbances that could contribute to disease progression and symptom exacerbation. Further research should aim to explore these relationships longitudinally, particularly in relation to treatment response and disease course.

### Limitations of the Study

The hematological study of patients in the locked wards was carried out with a very high degree of difficulty. The reason was that relationships and correlations were sought between the results of selected parameters in the peripheral blood of patients in the test and control groups. In a standard clinical procedure, developed and validated algorithms in psychiatry are used. Considering the problematic contact with the patient, it is essential to control hematological parameters, forecasting the manifestation of disease symptoms. There are few clinical studies of psychiatric patients in the literature. Hence, attempts were made to verify relationships and correlations in this experiment. The difficulties encountered were related to the test group. Patients with these diseases [Table 1] rarely cooperate in the therapeutic process. They are closed and disengaged and do not adhere to recommendations concerning proper nutrition, physical activity, and lifestyle. In general, hospitalization in locked wards is long-term, i.e., from a few to several months. According to algorithms, the attending physician’s role is reduced to controlling the patients’ behavior (aggression, dementia, etc.) through standard pharmacological therapy. In our study, we wanted to identify the nuances in hematological parameters that could forecast neurotic conditions, thus allowing us to respond appropriately by implementing appropriate therapy, diet, and supplementation. Another difficulty observed in the study was the hospital diet, which is classified by appropriate management in diseases mainly concerning metabolic disorders. According to current knowledge, a diet that ensures the provision of essential nutrients is not implemented.

Based on our observations, it can be concluded that an appropriate response with therapy, diet enrichment, and supplementation can mitigate the appearance of disease symptoms. However, such studies should be continued due to the difficulty of conducting studies in such a group of patients (number, age, mortality, contact with the patient). Despite the difficulty of conducting studies in such a group of patients (number, age, mortality, contact with the patient), and bearing in mind precisely these difficulties, we firmly believe such research calls for continuation.

## 5. Conclusions

The objective of this study was to identify hematological and biochemical correlations that may serve as early indicators of the manifestation and progression of psychiatric disorders in patients hospitalized in locked psychiatric wards. In this prospective observational study, blood samples from 28 patients with ICD-10 diagnoses (F03 and F06.2) were analyzed and compared with a control group of 10 individuals without psychiatric disorders. Blood samples were examined for selected hematological parameters—red blood cell fraction, white blood cell fraction (e.g., hemoglobin, hematocrit, and MCV), and nutritional biochemical markers (vitamin B12, vitamin D3, and glucose levels). The results showed significantly lower hemoglobin, hematocrit, and vitamin D3 and B12 levels in the patient group, suggesting risks of anemia and metabolic deficiencies.

This study highlights the importance of monitoring these parameters in routine care for psychiatric patients, which may aid in identifying deficiencies and implementing dietary and supplement interventions as support for traditional therapy. These findings provide new perspectives for further research on the role of hematological and biochemical parameters as potential biomarkers in psychiatry.

## Figures and Tables

**Table 1:** Classification of the diseases according to ICD10 [12].

ICD10	Diagnosis in Polish	Diagnosis in English	Diagnosis in Latin
F06.2	ORGANICZNE ZABURZENIA UROJENIOWE [PODOBNE DO SCHIZOFRENII]	NONE	SYNDROMA DELUSIONALE PSYCHO-ORGANICUM [SCHIZOPHRENIFORME].
F03	OTĘPIENIE BLIŻEJ NIE OKREŚLONE	ORGANIC, INCLUDING SYMPTOMATIC, MENTAL AND BEHAVIORAL DISORDERS. UNSPECIFIED DEMENTIA	DEMENTIA NON SPECIFICATA

Characteristics of the diseases according to ICD10 [12].

**Table 2:** Biochemical parameters were assessed in sick (N 10) and healthy (N 28) patients.

Test Results	Control Group			Test Group			
	Me	Q1	Q3	Me	Q1	Q3	*p* *
glucose	89.50	77.00	94.00	92.50	87.00	97.00	0.145
vit b12	418.50	251.00	532.00	278.00	166.50	401.50	0.026
vit D3	26.00	22.40	60.40	3.00	3.00	4.00	<0.001
leukocytes	6.60	5.40	7.45	5.94	5.35	6.71	0.288
erythrocytes	4.43	4.16	4.66	4.25d	3.90	4.50	0.082
hemoglobin	13.70	12.00	15.50	12.70	12.20	13.90	0.239
haematocrit	41.30	38.20	44.60	38.00	37.00	41.00	0.033
MCV	90.10	89.70	94.60	92.00	89.00	96.00	0.551
MCH	30.40	29.80	31.50	31.00	30.00	32.00	0.161
MCHC	33.10	32.70	33.60	34.00	33.00	34.00	0.132
RDW-SD	14.80	13.85	16.00	13.00	12.20	13.20	0.003
platelets	236.50	230.00	310.00	223.50	189.00	264.00	0.281
MPV	10.45	10.20	11.10	8.55	8.10	9.30	<0.001
neutrophils	59.65	48.90	65.20	52.50	47.00	56.00	0.093
lymphocytes	30.00	24.00	36.70	36.00	29.00	41.00	0.127
monocytes	7.75	7.00	9.90	7.00	7.00	8.00	0.078
eosinophils	2.25	1.30	3.30	4.00	3.00	5.00	0.098
basophils	0.50	0.30	0.70	1.00	0.00	1.00	0.268
neutrophils%	3.45	2.78	4.08	3.18	2.59	4.42	0.986
lymphocytes%	1.83	1.38	2.14	2.27	1.85	2.72	0.810
monocytes%	0.46	0.41	0.49	0.45	0.36	0.55	1.000
eosinophils%	0.11	0.09	0.20	0.22	0.15	0.29	0.012
basophils%	0.04	0.01	0.04	0.03	0.02	0.04	0.722

N = number of subjects in the test group; Me = median; Q1 and Q3 = first and third quartiles; *p* * Kruskal–Wallis test; as a statistically significant difference in the health of the patient value depending on the tested morphological and biochemical parameters.

**Table 3:** The anthropometric characteristics of the groups subjected to the study.

Parameters	Control Group			Test Group			*p* *
	Me	Q1	Q3	Me	Q1	Q3	
Age [year]	42.50	38.00	64.00	70.50	67.50	76.00	0.001
High [cm]	169.00	166.00	172.00	156.50	151.50	161.50	0.003
Weight [kg]	71.50	63.00	86.00	59.90	53.80	72.50	0.031
BMI [kg/m^2^]	25.71	22.14	28.13	24.35	22.02	28.85	0.705

Data are expressed as mean ± SD; BMI = body mass index, Me = median; Q1 and Q3 = first and third quartiles; *p* * Kruskal–Wallis test.

**Table 4:** Comparison of age and sex of the patients studied in the context of weight, BMI, glycemia, HGB, HCT, and PLT.

Parameters	Vit. B12	Vit. D	Waist Circumference	Weight	BMI	Glycemia	HGB	HCT	PLT
Age	0.449 *	0.393	0.391	1.000	0.509	0.223	0.549	0.352	0.272
Sex	0.751	0.223	0.433	0.293	0.827	0.557	0.195	0.429	0.403

* chi-square test.

## Data Availability

Data Availability Statements are available in the corresponding author due to GDPR—The General Data Protection Regulation.

## Supplementary Materials

The following supporting information can be downloaded at https://www.mdpi.com/article/10.3390/nu17060959/s1, Table S1: Reference ranges of hematological parameters.
