# Supplementary material for: Hematological Correlations as Predictors of Disease Manifestations in Psychiatric Inpatients
Source: Nutrients. 2025 Mar 10;17(6):959. doi: 10.3390/nu17060959 (PMC11944345; doi:10.3390/nu17060959)
Supplement: Supplementary file 1 [file nutrients-17-00959-s001.zip › nutrients-3474978-supplementary.pdf]

Table S1. Reference ranges of hematological parameters after Tomaszewski[13]

No	Name of parameter [unit]	Reference range
1.	Leukocytes - WBC [10^3/mm^3].	4.5-10.0
2.	Erythrocytes - RBC [10^6/mm^3].	3.7-5.8
3.	Hemoglobin - HGB [g/dl].	12.6-17.4
4.	Hematocrit - HCT [%].	36-52
5.	MCV [um ^3].	80-98
6.	MCH [pg]	28-33
7.	MCHC [g/dl]	32-36
8.	RDW-SD [%]	11.5-14.5
9.	Platelets - PLT [10^3/mm^3].	150-400
10.	MPV [um ^3].	6.0-10.0
11.	Neutrophils - NEU [%].	45-70
12.	Lymphocytes - LYM [%].	20-45
13.	Monocytes - MON [%]	3-10
14.	Eosinophils - EOS [%].	0-5
15.	Basophils - BAS [%]	0-5
16.	Neutrophils -NEU#	1.80-7.0
17.	Lymphocytes -LYM#	0.8-4.5
18.	Monocytes -MON#	0.1-1.0
19.	Eosinophils -EOS#	0.00-0.40
20.	Bazophiles -BAS#	0,00-0,20
21.	Glucose/glycaemia [mg/dL].	70-99
22.	Vitamin D3 [pg/mL].	<20 pg/mL,- deficiency;<br>20-29 pg/mL, - insufficient;<br>30-100 pg/mL, - sufficient;<br>>100 pg/mL, - potentially toxic;
23.	Vitamin B12 [ng/mL].	197-771

Own elaboration based on Tomaszewski[13].
